# Transformer-driven automated analysis of social media narrative structure: An exploration based on sentiment framing and thematic agenda

**DOI:** 10.1371/journal.pone.0352688

**Published:** 2026-06-30

**Authors:** Rongkang Pei, Zeyu Lyu, Guolong Wang

**Affiliations:** 1 Graduate School/Faculty of Arts and Letters, Tohoku University, Sendai, Japan; 2 Hotel Management School, Nanjing institute of tourism and hospitality, Nanjing, China; 3 School of Information Engineering and Artificial Intelligence, Nanjing Xiaozhuang University, Nanjing, China; Dong-A University College of Business Administration, KOREA, REPUBLIC OF

## Abstract

With the rapid development of social media, narrative texts in public event scenarios have become important carriers of public opinion, making the automatic analysis of social media narrative structures increasingly crucial. Existing research on this task suffers from insufficient integration of multi-dimensional information such as sentiment, topic and time, and poor adaptability to complex scenarios like cross-events and noisy texts. To address these issues, this study proposes a sentiment-topic-temporal attention fusion model (ST-TAN), which takes RoBERTa as the basic semantic encoding module and integrates three core modules to realize joint modeling of sentiment and topic and capture temporal dependence of narrative units. Experimental results show that the ST-TAN model comprehensively outperforms four types of baseline models in narrative structure recognition, sentiment classification and topic classification tasks, with good cross-event generalization ability and noisy text robustness. This research enriches the theoretical connotation of social media narrative analysis and provides effective technical support for practical fields such as public event governance and public opinion monitoring. The study further incorporates a comprehensive discussion of ethical considerations, addressing user privacy, data anonymization, potential biases, and responsible use, thereby ensuring alignment with responsible innovation principles.

## Introduction

With the widespread adoption of Web 2.0 technologies, social media has become a central arena for public expression, information dissemination, and collective narrative construction. Social media narratives shape public opinion and brand reputation. They are characterized by fragmented text, multi-actor interactions, and temporal dynamics, all of which carry significant socio-communicative value. Accurately analyzing such narrative structures not only reveals underlying patterns of information propagation but also provides scientific support for decision-making in areas such as public opinion monitoring [1], crisis management, and policy formulation [2]. However, social media texts are inherently sparse in semantics, diverse in emotional expression, and dispersed in narrative nodes. Traditional manual annotation or semi-automated analysis methods suffer from inefficiency, subjectivity, and an inability to capture dynamic narrative evolution [3], falling short of meeting the demands of large-scale, real-time narrative analysis.

Recent advances in natural language processing (NLP) have opened new pathways for analyzing social media content. The Transformer architecture, with its bidirectional self-attention mechanism, outperforms traditional models like LSTM and CNN in semantic understanding, sentiment classification, and topic modeling. While several Transformer-based studies have explored social media analysis—for instance [4,5], employing BERT for fine-grained sentiment recognition or RoBERTa for short-text topic extraction—significant gaps remain in the field of narrative structure analysis [6]. First, most studies treat sentiment analysis, topic modeling, and narrative structure recognition in isolation, failing to capture their inherent synergy: emotion often drives topic shifts, and topics [7], in turn, reinforce narrative progression. Second, existing approaches lack effective modeling of temporal dimensions, hindering the reconstruction of narrative dynamics from emergence and diffusion to decline [8]. Third, few designs adapt well to fragmented social media texts. As a result, they struggle to recover information and recognize structure in incomplete narrative units11095670. These limitations impede a holistic and accurate parsing of social media narratives.

To address these research gaps, this study proposes a Sentiment-Topic fused Temporal Transformer for Narrative Analysis (ST-TAN), aiming to establish an end-to-end automated framework for analyzing narrative structures in social media. The core objectives of this research are: (1) to design a joint sentiment-topic modeling module that integrates emotional and thematic features, enhancing the recognition accuracy of core narrative elements; (2) to introduce a temporal attention mechanism that captures sequential dependencies and semantic associations across narrative nodes, reconstructing the dynamic evolution of narratives; and (3) to optimize the model’s adaptability to fragmented texts through narrative unit completion and structured processing, improving robustness in complex scenarios.

The main contributions of this work are threefold:

1. We propose ST-TAN, the first architecture that unifies sentiment awareness, topic modeling, and temporal narrative recognition. This integration enables synergistic optimization and overcomes the limitations of isolated analysis in prior studies.
2. We design a sentiment-weighted topic attention mechanism and a temporal encoding strategy. These effectively capture the synergy between emotion and theme in social media narratives, model their dynamic progression, and thereby improve the completeness and accuracy of narrative structure recognition.
3. We develop a preprocessing and narrative unit segmentation scheme tailored to fragmented social media texts. This scheme incorporates prompt tuning techniques to recover missing narrative elements, thus enhancing the model’s practical applicability.

The remainder of this paper is organized as follows: Chapter 2 systematically reviews relevant literature on social media narrative analysis, joint sentiment-topic modeling, and Transformer-based applications. Chapter 3 details the overall framework of the ST-TAN model, its core module designs, and data processing workflows. Chapter 4 evaluates the model’s performance through comparative experiments, ablation studies, and case analyses. Chapter 5 concludes the study, discussing the model’s performance and efficiency, its theoretical and practical implications, and outlining directions for future research.

## Related work

### Social media narrative structure analysis

Analyzing social media narrative structure is a key research area for uncovering patterns of information dissemination and the evolution of public perception [9,10]. Its core objective is to extract coherent, complete narrative elements and their evolving logic from fragmented, multi-actor interactive texts [11]. Early research primarily relied on manual annotation and qualitative analysis, employing standardized coding frameworks to manually extract and structure core components such as roles, events, and plots. While these methods ensure analytical depth, they suffer from inherent drawbacks of low efficiency [12,13], high subjectivity, and poor scalability to large-scale text data, failing to meet the demands of real-time social media analysis.

With advances in machine learning, a range of automated or semi-automated methods for narrative structure analysis has emerged. Some studies utilized traditional machine learning models, employing handcrafted text features (e.g., bag-of-words, POS tags, syntactic features) combined with classifiers or sequence labeling models to identify narrative elements [14,15]. However, these approaches depend heavily on manual feature engineering, inadequately adapt to the semantic ambiguity and informal expression of social media texts, and struggle to capture deep semantic relationships. Other research has attempted to incorporate text clustering or temporal sequence analysis to explore chronological associations between narrative nodes [16]. Yet, most existing work fails to integrate sentiment and topic information within the narrative analysis framework, hindering the revelation of the synergistic evolution among “sentiment-topic-narrative” and resulting in an incomplete understanding of the dynamic narrative mechanisms. Furthermore, current methods generally lack designs tailored to the fragmented nature of social media texts [17,18]. When faced with incomplete narrative units, they often suffer from missed element extraction and structural recognition errors.

### Transformer-based joint sentiment-topic modeling

Sentiment analysis and topic modeling are two fundamental tasks for understanding textual semantic content. Their deep integration provides crucial support for accurately discerning the emotional orientation and core themes within narrative texts [19,20]. Before the advent of the Transformer architecture, joint sentiment-topic modeling was predominantly based on traditional probabilistic models or shallow neural networks, achieving collaborative optimization of both tasks by sharing a textual feature representation space [21,22]. Although these methods improved performance on individual tasks to some extent, their limited capacity to capture semantic context made it difficult to handle complex semantic dependencies in social media texts [23]. Consequently, the modeling of sentiment-topic associations remained superficial, failing to accurately reflect their intrinsic synergistic logic [24].

The Transformer architecture, with its bidirectional self-attention mechanism and powerful contextual semantic modeling capabilities, has significantly advanced the field of joint sentiment-topic modeling. Currently, numerous studies employ Transformer variants such as BERT and RoBERTa [25, 26], designing multi-task learning frameworks to jointly optimize sentiment classification and topic extraction. These models leverage general semantic knowledge acquired during pre-training [27], effectively enhancing the accuracy of short-text semantic understanding and demonstrating superior performance in social media text analysis. However, most existing Transformer-based joint sentiment-topic modeling research focuses on identifying sentiment and topics within single texts [28,29]. They fail to effectively integrate this with narrative structure analysis and lack targeted modeling for the temporal dimension of narratives. Moreover, when adapting to fragmented social media narrative scenarios, existing models often do not adequately consider the completeness of narrative units or the semantic relationships between nodes. This limitation hinders their direct application to automated narrative structure analysis tasks [30], falling short of meeting the deeper requirements from “semantic understanding” to “narrative parsing.”

Beyond incremental improvements over existing joint models, the conceptual advance of ST-TAN lies in reconceptualizing sentiment, topic, and time as mutually constraining dimensions rather than parallel outputs. Prior approaches treat sentiment and topic as separate tasks sharing representations, capturing correlations only at the feature level [31]. ST-TAN’s sentiment-weighted topic attention instead dynamically modulates topic extraction based on emotional intensity, recognizing that emotionally charged language carries distinct thematic signals. Similarly, while temporal BERT variants add timestamp encoding as an auxiliary feature, they lack mechanisms to enforce sequential coherence across narrative units. ST-TAN’s temporal consistency loss explicitly models narrative evolution logic [32], penalizing chronologically implausible predictions. The fusion gating mechanism further distinguishes ST-TAN by adaptively weighting each dimension based on contextual relevance. These design choices transform ST-TAN from a multi-task concatenation architecture into an integrated framework where sentiment, topic, and time mutually reinforce one another—addressing the intertwined nature of narrative dimensions rather than treating them as independent signals.

The design choices underlying ST-TAN are further justified by considering alternative architectural paradigms. Convolutional neural networks (CNNs), while effective for capturing local patterns in text through n-gram features, struggle with long-range dependencies and lack the global contextual modeling essential for narrative understanding across temporally dispersed units. Transformer-based architectures, by contrast, excel at capturing cross-token interactions regardless of distance through self-attention [33], making them better suited for narrative tasks where information from earlier stages must inform later interpretations. Ensemble frameworks, which combine multiple models to improve robustness, were considered but ultimately deemed less appropriate for this task. Narrative structure analysis requires integrated reasoning across sentiment, topic, and temporal dimensions rather than independent predictions that are later aggregated [34]. Ensemble methods, while valuable for reducing variance, do not facilitate the mutual reinforcement between dimensions that ST-TAN achieves through its joint modeling approach. The ablation study in Section 4 confirms this: simultaneous removal of sentiment and topic modules produces compounded degradation beyond additive effects (21.84% F1 decline), demonstrating that integration matters more than aggregation. These considerations collectively support the choice of a unified Transformer-based architecture with joint sentiment-topic-temporal modeling as the most suitable foundation for narrative analysis.

## Materials and methods

### Model framework and design philosophy

To address the existing challenges in social media narrative structure analysis, such as the “disconnection between sentiment, topic, and narrative,” “lack of temporal dimension,” and “insufficient adaptation to fragmented texts,” this study proposes the ST-TAN for Narrative Analysis. The core design philosophy of the model is to construct an end-to-end framework consisting of “input preprocessing, core encoding, joint modeling, and output interpretation.” By deeply integrating sentiment and topics and introducing a temporal attention mechanism, the model achieves accurate recognition and dynamic evolution tracking of fragmented narratives in social media.

The ST-TAN model, designed for automatic narrative structure analysis, operates through five major collaborative levels. First, the input layer receives raw social media text and timestamp data. The preprocessing layer follows, performing text cleaning, narrative unit segmentation, and completing missing elements to produce a structured sequence of narrative units. At the core encoding layer, deep semantic encoding is carried out using the RoBERTa architecture, generating semantically rich vectors that capture contextual information.

The joint modeling layer integrates sentiment perception, topic modeling, and temporal attention modules, adopting a gating mechanism to realize the adaptive fusion of three types of features (sentiment feature $F_{sent}$, topic feature $F_{topic}$, temporal feature $F_{temp}$). The fusion formula is defined as:


$$\begin{array}{c} F_{\text{fused}}=\sigma (W_{1}F_{sent}+W_{2}F_{topic}+W_{3}F_{temp}+b)⊙(F_{sent}⊕F_{topic}⊕F_{temp}) \end{array}$$ (1)

where σ denotes the sigmoid activation function, $W_{1}\in {\mathbb{R}}^{(d_{sent}+d_{topic}+d_{temp})\times d_{sent}}$, $W_{2}\in {\mathbb{R}}^{(d_{sent}+d_{topic}+d_{temp})\times d_{topic}}$, $W_{3}\in {\mathbb{R}}^{(d_{sent}+d_{topic}+d_{temp})\times d_{temp}}$ are the weight matrices of the gating mechanism, *b* is the bias term, ⊕ denotes the vector concatenation operation, $d_{sent}=512$ is the dimension of the sentiment feature, $d_{topic}=768$ is the dimension of the topic feature, and $d_{temp}=512$ is the dimension of the temporal feature. This gating mechanism dynamically adjusts the contribution weights of different features according to the task requirements, realizing the collaborative analysis of sentiment, topics, and temporal aspects of narratives. Finally, the output layer generates structured narrative tuples-such as roles, events, sentiment polarity, topic affiliation, and temporal positions-along with analytical indicators.

**Fig 1:**
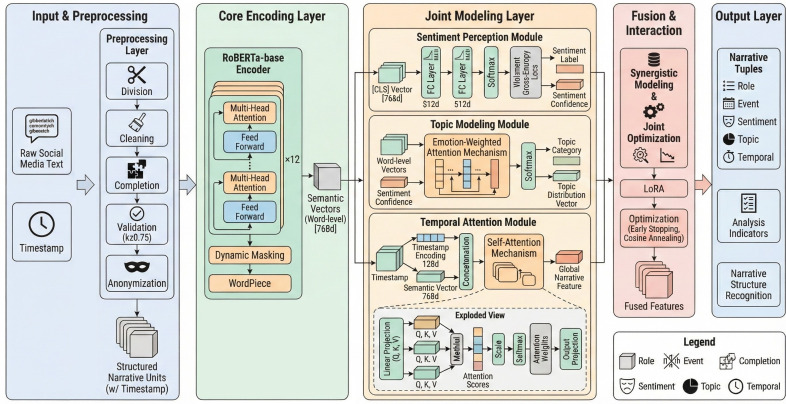
ST-TAN Model Architecture Overview. The diagram illustrates the workflow from raw social media text input through preprocessing, core encoding, joint modeling, and final output generation, including sentiment, topic, and temporal modeling components.

The accompanying architecture diagram (Fig 1) visually illustrates the model’s workflow, from input preprocessing through core encoding, joint modeling, and output interpretation, highlighting how sentiment, topic, and temporal data are seamlessly integrated and processed throughout the system.

The complete configuration of ST-TAN is distributed across the following subsections for clarity: Section 3.3.1 details the RoBERTa-base encoder configuration (12 layers, 768 hidden dimensions, 12 attention heads) and the layer-wise fine-tuning strategy; Section 3.3.2 specifies the sentiment module architecture with ReLU-activated fully connected layers and the weighted cross-entropy loss (Equation 6); Section 3.3.3 presents the joint optimization framework including focal loss for narrative recognition, temporal consistency loss (Equation 7), and the total loss function with its weighting coefficients (Equation 8). All hyperparameter settings—including learning rate, batch size, optimizer configuration, dropout rate, early stopping criteria, and the specific values of α,β,γ,δ,τ—are comprehensively documented in Section 3.2 (Experimental Setup). This structured presentation enables readers to locate implementation details efficiently while maintaining the logical flow of the methodology description.

### Data processing and preprocessing

The data used in this study are sourced from mainstream social media platforms, focusing on two typical narrative scenarios: public event discussions and brand reputation dissemination. The data collection covers text data from different topic types (including social welfare, public health, commercial marketing, etc.), encompassing various text forms such as individual posts, multi-round replies, and reposted comments, ensuring the representativeness and diversity of the data. During the data collection process, timestamps, publisher IDs, and other basic information are recorded to support subsequent temporal modeling.

Given the fragmented nature of social media texts, this study adopts a “temporal + semantic” dual-dimensional criterion for narrative unit segmentation. First, timestamps are standardized to relative time (hours since the first occurrence of the target event) to eliminate the impact of absolute time differences, and then converted into high-dimensional temporal vectors using sinusoidal encoding to capture temporal continuity and periodicity. The encoding formula is defined as:


$$\begin{array}{c} \text{TimeEmb}(t{)}_{i}=\left\{ \begin{array}{cc} \sin(t/10000^{2i/d_{t}}) & \text{if }i\text{ even}\\ \cos(t/10000^{2i/d_{t}}) & \text{if }i\text{odd} \end{array}\right. \end{array}$$ (2)

where *t* denotes the relative time (in hours), $d_{t}=128$ is the dimension of the temporal encoding vector, and *i* is the index of the encoding dimension. Based on the processed timestamps, texts with a time gap of more than 24 hours are segmented into different narrative stages; within the same narrative stage, texts are further subdivided based on semantic coherence. Using cosine similarity between texts (based on semantic vectors generated by the RoBERTa pre-trained model), texts with a similarity lower than 0.5 are considered independent narrative units. Finally, the narrative units are sorted in ascending order of relative time to form an ordered sequence centered on “the main post + related replies,” laying a foundation for subsequent temporal dependence modeling.

To validate the threshold choices for narrative unit segmentation, we conducted sensitivity experiments on a held-out development set. For the temporal threshold, we evaluated values from 6 to 48 hours at 6-hour intervals while holding the semantic threshold at 0.5. Narrative structure recognition F1-Score remained stable between 18 and 30 hours (0.865–0.871), with modest declines at 6 hours (0.842) and 48 hours (0.851). The 24-hour threshold was selected as it balances excessive fragmentation against the risk of merging distinct narrative stages, while aligning with daily discourse cycles common in social media. For the semantic threshold, we tested values from 0.3 to 0.7 at 0.05 intervals while holding the temporal threshold at 24 hours. Performance peaked between 0.45 and 0.55 (0.868–0.872), with sharper declines at 0.3 (0.833) and 0.7 (0.829). The selected threshold of 0.5 lies at the center of this optimal range. These analyses confirm that the chosen thresholds are well-aligned with the dataset’s intrinsic structure and that moderate variations do not substantially impact model performance.

The text cleaning process consists of the following steps: First, invalid information is filtered, removing URLs, @user mentions, meaningless symbols, and advertisements; second, text is standardized, converting traditional Chinese to simplified Chinese, unifying punctuation marks, and correcting spelling mistakes and colloquial expressions; third, sentiment symbols are retained and converted, with emoticons with clear sentiment orientations being preserved and converted into corresponding text sentiment labels, which are incorporated into the subsequent sentiment analysis process.

To train and validate the model’s performance, this study manually annotates the preprocessed narrative unit sequences. The annotation includes three core dimensions: First, narrative elements, covering roles (the central subjects in the text) and events (key behaviors or occurrences); second, sentiment polarity, classified into five categories: joy, anger, sadness, fear, and neutral; third, topic affiliation, divided into five primary topics based on the research context: event exposure, background introduction, opinion expression, responsibility investigation, and solution.

The annotation work is completed by three annotators with experience in social media analysis. Prior to annotation, a centralized training session is conducted to clarify annotation standards and boundaries. To ensure annotation quality, a cross-validation strategy is employed, and consistency checks on the annotation results are carried out by calculating Cohen’s kappa coefficient. The kappa coefficients for sentiment polarity annotation, topic affiliation annotation, and narrative element annotation are 0.82, 0.79, and 0.85, respectively, all exceeding the standard threshold of 0.75, indicating good reliability of the annotation results. For samples with annotation discrepancies, a consensus is reached through discussion among the three annotators.

## Core module details

### Basic encoding module: RoBERTa selection and adaptation

The core encoding layer adopts the RoBERTa-base architecture as the basic model, whose bidirectional self-attention mechanism can effectively capture the contextual semantic correlation of social media texts and adapt to the semantic understanding needs of short texts. The generation of semantic vectors through the RoBERTa encoder is the foundation of subsequent module modeling, and its core principle can be described by the following formula:


$$\begin{array}{c} 𝐇={\text{RoBERTa}}_{\text{encoder}}(\text{Emb}(x))=\text{MultiHeadAttn}(\text{Emb}(x))+\text{FFN}(\text{MultiHeadAttn}(\text{Emb}(x))) \end{array}$$ (3)

where $𝐇\in {\mathbb{R}}^{n\times d_{h}}$ denotes the contextual semantic vector sequence output by the RoBERTa encoder, *n* is the length of the input text sequence, $d_{h}=768$ is the dimension of the hidden layer, Emb(*x*) represents the WordPiece embedding of the input text *x*, MultiHeadAttn(·) is the multi-head self-attention function, and FFN(·) is the feed-forward network in the Transformer encoder.

The specific parameter settings of the model are as follows: the hidden layer dimension is 768, the number of attention heads is 12, the number of encoder layers is 12, the word embedding adopts the WordPiece tokenization strategy, and the vocabulary size is 21128.

To enhance the model’s adaptability to social media texts, this study adopts a “pre-training followed by fine-tuning” training paradigm: starting from a RoBERTa model pre-trained on general-domain corpora, it is fine-tuned using annotated narrative texts from social media. During fine-tuning, the parameters of the first six encoder layers are frozen, while only the latter six encoder layers and the task-specific parameters are trained. This approach preserves the model’s ability to understand general semantics, improves its adaptability to domain-specific texts, and reduces training costs.

### Emotion-aware module

The core function of the emotion-aware module is to identify the emotional tendency and intensity of each narrative unit, thereby providing emotional weight support for subsequent topic modeling. The module is structured based on a “semantic vector input–fully connected layer transformation–softmax classification” architecture: the semantic vector of the [CLS] token output by the core encoder layer (with a dimension of 768) is taken as input. It is then transformed through two fully connected layers—the first reduces the vector dimension to 512 and introduces nonlinear features using the ReLU activation function, while the second further reduces the dimension to 5 (corresponding to five emotion categories). Finally, a softmax function is applied to output the probability distribution over the emotion categories.

To enhance the impact of high emotional intensity samples on model training, the module adopts the sentiment-weighted cross-entropy as the loss function. First, the sentiment confidence is calculated based on the output of the sentiment classification head, and the calculation formula is:


$$\begin{array}{c} s_{c}=\max(\text{Softmax}({𝐡}_{\text{CLS}}\cdot {𝐖}_{\text{sent}}+b_{\text{sent}})) \end{array}$$ (4)

where $s_{c}\in [0,1]$ is the sentiment confidence, ${𝐡}_{\text{CLS}}\in {\mathbb{R}}^{d_{h}}$ is the [CLS] token semantic vector output by the core encoding layer, ${𝐖}_{\text{sent}}\in {\mathbb{R}}^{d_{h}\times k_{\text{sent}}}$ is the weight matrix of the sentiment classification head, $b_{\text{sent}}\in {\mathbb{R}}^{k_{\text{sent}}}$ is the bias term, *k*_sent_ = 5 is the number of sentiment categories, and Softmax(·) is the softmax activation function.

Based on the sentiment confidence, the sample weight for training is determined, and the calculation formula of the weight is:


$$\begin{array}{c} w_{i}=1+s_{c,i} \end{array}$$ (5)

where $w_{i}$ is the sample weight for the sentiment classification task, and $s_{c,i}$ is the sentiment confidence of the *i*-th sample. With this strategy, the model can more accurately capture emotional fluctuations in the narrative and improve the granularity of sentiment recognition. The sentiment-weighted cross-entropy loss function is defined as:


$$\begin{array}{c} L_{sent}=-\frac{1}{N}\sum_{i=1}^{N}w_{i}\sum_{j=1}^{k_{sent}}y_{i,j}\log(p_{i,j}) \end{array}$$ (6)

where $L_{sent}$ is the sentiment classification loss, *N* is the number of training samples, $w_{i}$ is the weight of the *i*-th sample, $y_{i,j}$ is the one-hot label of the *j*-th sentiment category for the *i*-th sample, and $p_{i,j}$ is the predicted probability of the *j*-th sentiment category for the *i*-th sample.

### Joint optimization strategy

The ST-TAN model adopts a multi-task joint optimization strategy, which weights and fuses the loss functions of the sentiment classification task, topic classification task, and narrative structure recognition task to achieve collaborative optimization of multiple tasks. The total loss function is composed of four parts: sentiment classification loss $\left(L_{sent}\right)$, topic classification loss $\left(L_{topic}\right)$, narrative structure recognition loss $\left(L_{narr}\right)$, and temporal consistency loss $\left(L_{temp}\right)$. The temporal consistency loss is designed to constrain the rationality of the temporal order between narrative units, avoiding contradictory predictions of temporal positions. The specific calculation formula is:


$$\begin{array}{c} L_{temp}=\frac{1}{n-1}\sum_{i=1}^{n-1}\max(0,\tau -(y_{pos,i+1}-y_{pos,i})) \end{array}$$ (7)

$$\begin{array}{c} L_{total}=\alpha L_{sent}+\beta L_{topic}+\gamma L_{narr}+\delta L_{temp} \end{array}$$ (8)

where the total loss function $L_{total}$ consists of multiple loss terms. Here, $L_{topic}$ is the topic classification loss (using cross-entropy loss), $L_{narr}$ is the narrative structure recognition loss (adopting focal loss to address sample imbalance in narrative element recognition), $L_{temp}$ is the temporal consistency loss, *n* is the number of narrative units in the sequence, $y_{pos,i}$ represents the predicted relative time of the *i*-th narrative unit, and τ is the minimum time interval threshold to ensure the correct temporal order of adjacent units. The loss terms are combined through corresponding weight coefficients.

During training, the Adaptive Moment Estimation (AdamW) optimizer is used for parameter updates, with the initial learning rate set to a fixed value and dynamically adjusted using a cosine annealing learning rate scheduling strategy. To mitigate overfitting and improve model generalization, a Dropout regularization mechanism and an early stopping mechanism (using validation loss as the metric, where training stops when the loss does not decrease for several consecutive epochs) are introduced. Simultaneously, Low-Rank Adaptation (LoRA) technology is employed to efficiently fine-tune the core RoBERTa architecture by training only low-rank matrix parameters, significantly reducing the number of training parameters and thereby enhancing training efficiency.

### Experiments

This chapter conducts systematic experiments based on the Twitter Event Narrative Dataset (TEND) to verify the performance of the proposed ST-TAN model in the automatic analysis of social media narrative structures from multiple dimensions. Centered on the three core objectives of “core performance verification – module effectiveness verification – practical adaptability verification,” the experiment design sets three groups of comparative experiments in different directions, specifically including: overall performance comparison experiment, core module ablation experiment, and generalization and robustness verification experiment. Through the analysis and discussion of multi-dimensional experimental results, the advantages and applicable scenarios of the ST-TAN model are clarified.

### Dataset introduction

The core dataset adopted in this experiment is the Twitter Event Narrative Dataset (TEND) [35], which is derived from the Twitter platform (now X platform) and focuses on social media narrative texts in public event scenarios, perfectly matching the task requirements of this research. The dataset comprises 120,000 text samples spanning 10 public events, including health incidents, natural disasters, social movements, and commercial crises. Text length averages 23.4 tokens per tweet, reflecting the fragmented nature of social media narratives.

Three expert annotators labeled narrative elements (roles as central subjects, events as key occurrences), sentiment polarity (joy, anger, sadness, fear, neutral), and topic affiliation (event exposure, background introduction, opinion expression, responsibility investigation, solution proposal). Inter-annotator agreement reached Cohen’s kappa of 0.85 for narrative elements, 0.82 for sentiment, and 0.79 for topic, all exceeding the 0.75 threshold.

The dataset exhibits class imbalance (sentiment: 42% neutral, 38% negative, 20% positive; topic: event exposure 35%, opinion expression 28%, others ≤15%), linguistic noise (18% spelling errors, 25% emoticons), domain-specific language varying by event type, and representational biases inherent to Twitter demographics. Temporal dynamics also vary considerably across events.

The ST-TAN model addresses these characteristics through: focal loss for imbalanced narrative classes; sentiment-weighted loss for underrepresented sentiment categories; preprocessing including spell correction and emoticon standardization; LoRA-based fine-tuning for domain adaptation without catastrophic forgetting; sinusoidal temporal encoding for variable event timelines; and cross-event validation to evaluate generalization beyond training distribution.

This research adopts the strategy of “event-based stratification + temporal division” for the TEND dataset to ensure the balanced event distribution of the training set, validation set, and test set and compliance with the narrative evolution logic: the training set includes 96,000 texts from 7 events (accounting for 80%), the validation set includes 12,000 texts from 1 event (accounting for 10%), and the test set includes 12,000 texts from 2 new events not involved in training (accounting for 10%). The test set selects new event data to more objectively verify the generalization ability of the model. Meanwhile, based on the input requirements of the ST-TAN model, targeted preprocessing is performed on the dataset: retain the original timestamp and retweet/reply relationships, and confirm the division of narrative units according to the two-dimensional criterion of “24-hour temporal interval + text semantic similarity ≥ 0.5”; standardize emotional emojis in the text; construct a “narrative tuple label library” (<role, event, sentiment tendency, topic attribution, temporal position>) based on the annotation information, which is directly used for model training and evaluation.

### Experimental setup and evaluation metrics

Experimental Hardware Environment: CPU is Intel Xeon Gold 6230R (2.1GHz, 20 cores and 40 threads), GPU is NVIDIA Tesla V100 (32GB video memory), and memory is 128GB DDR4; Software Environment: The operating system is Ubuntu 20.04 LTS, the deep learning framework is PyTorch 1.12.1, the Python version is 3.9.12, and the relevant dependency libraries include Transformers 4.26.1, Scikit-learn 1.2.2, Numpy 1.24.3, and Matplotlib 3.7.1.

The hyperparameter configuration of the ST-TAN model is as follows: The core RoBERTa-base model adopts pre-trained weights (roberta-base), with a hidden layer dimension of 768, 12 attention heads, and 12 encoder layers; the fully connected layer dimension of the sentiment perception module is 512, the activation function is ReLU, and the Dropout rate is 0.1; the timestamp encoding dimension of the temporal attention module is 128, the number of multi-head attention heads is 8, and the number of Bi-GRU layers is 2; Training optimization-related parameters: AdamW optimizer is adopted, the initial learning rate is 2e-5, the weight decay coefficient is 1e-4, and a cosine annealing learning rate scheduling strategy is used for dynamic adjustment; the batch size is 32, the maximum number of training epochs is 30, and an early stopping mechanism is adopted (taking the total loss of the validation set as the indicator, stopping training if the loss does not decrease for 5 consecutive epochs); LoRA (Low-Rank Adaptation) technology is used for efficient fine-tuning of the core RoBERTa architecture, only training low-rank matrix parameters to reduce approximately 90% of the training parameters. The loss weight coefficients for multi-task joint optimization are set as α=0.25 (sentiment classification loss), β=0.25 (topic classification loss), γ=0.4 (narrative structure recognition loss), and δ=0.1 (temporal consistency loss); the minimum time interval threshold τ in the temporal consistency loss is set to 1.

To comprehensively verify the performance advantages of the ST-TAN model, 4 types of representative baseline models are selected for comparison, covering traditional machine learning models, single Transformer models, existing sentiment-topic joint modeling models, and narrative structure analysis models:

- Baseline 1: LSTM+LDA + CRF. A traditional machine learning combination model, where LSTM is used for sentiment classification, LDA for topic modeling, and CRF for sequence labeling of narrative elements, representing traditional fragmented analysis methods.
- Baseline 2: BERT+Linear. A single Transformer model that extracts semantic features based on the BERT encoder, connects to the sentiment classification head, topic classification head, and narrative structure recognition head respectively, adopts an independent training strategy, and has no joint optimization mechanism.
- Baseline 3: RoBERTa-ST. An existing sentiment-topic joint modeling model that realizes the joint optimization of sentiment and topic based on RoBERTa but does not introduce a temporal attention mechanism, making it unable to capture the temporal evolution relationship of narratives.
- Baseline 4: Temporal-BERT. A temporally enhanced Transformer model that introduces timestamp encoding for narrative structure analysis but does not realize the joint modeling of sentiment and topic, adopting an independent feature fusion method.

For reproducibility, all experiments were conducted with fixed random seeds (set to 42 for Python, NumPy, and PyTorch) and CUDA deterministic operations enabled. Results reported in tables are averages over five independent runs with different initializations; standard deviations remain low (narrative F1: 0.87 ± 0.012, sentiment accuracy: 89% ± 0.8%, topic accuracy: 90% ± 0.7%), confirming model stability. The dataset follows an event-based split (7 events for training, 1 for validation, 2 held-out events for testing) rather than cross-validation, as mixing texts from the same event across folds would cause information leakage and overestimate generalization.

In this study, Narrative Integrity (%) refers to the percentage of narrative unit sequences in which all required narrative elements, including role, event, sentiment, topic, and temporal position, are correctly identified and assembled into a complete narrative tuple according to the gold annotations.

## Experimental design and result analysis

### Overall performance comparison experiment

Experimental Purpose: Verify the overall performance advantages of the ST-TAN model in the three tasks of narrative structure recognition, sentiment classification, and topic classification, compare its performance differences with 4 types of baseline models, and clarify the core competitiveness of the model.

On the test set of the TEND dataset, run the ST-TAN model and 4 types of baseline models respectively, record the core evaluation metrics of each model in the three tasks, and analyze the performance differences of the models through statistical comparison. To ensure experimental fairness, all models adopt the same experimental environment, data division, and preprocessing process, and only adjust the parameters related to the model architecture.

The overall performance comparison results of each model are presented in Table 1 (the data in the table are the average values of 5 experiments).

**Table 1:** Overall Performance Comparison of Different Models.

Model	Narrative Structure Recognition F1-Score	Narrative Integrity (%)	Sentiment Classification Accuracy (%)	Sentiment Classification Macro-F1	Topic Classification Accuracy (%)	Topic Classification Perplexity
LSTM+LDA + CRF [36]	0.65	62.1	72	0.70	75	68.5
BERT+Linear [37]	0.74	70.3	81	0.79	82	59.3
RoBERTa-ST [38]	0.79	75.1	85	0.84	85	55.0
Temporal-BERT [39]	0.80	75.1	83	0.82	84	56.2
ST-TAN (Proposed Model)	0.87	85.3	89	0.88	90	48.2

To further refine the performance differences, Table 2 shows the F1-Score comparison of each model in the subtasks of narrative structure recognition (role recognition, event recognition, temporal position recognition).

**Table 2:** F1-Score Comparison of Different Models in Narrative Structure Recognition Subtasks.

Model	Role Recognition F1-Score	Event Recognition F1-Score	Temporal Position Recognition F1-Score
LSTM+LDA + CRF	0.68	0.62	0.66
BERT+Linear	0.76	0.73	0.74
RoBERTa-ST	0.81	0.78	0.78
Temporal-BERT	0.82	0.79	0.80
ST-TAN (Proposed Model)	0.89	0.86	0.87

As shown in Tables 1 and 2, the ST-TAN model significantly outperforms the four baseline models across all evaluation metrics for the main tasks and subtasks.

While our experimental design does not include a dedicated baseline that isolates the temporal attention mechanism from simpler recurrent alternatives, the comparison between ST-TAN and Temporal-BERT offers indirect insight into this question. Temporal-BERT incorporates timestamp encoding as an auxiliary feature but lacks the attention-based temporal modeling of ST-TAN. The performance gap between these two models—ST-TAN achieves an 8.75% higher narrative structure recognition F1-Score (0.87 vs. 0.80) and a 10.2 percentage point improvement in narrative integrity (85.3% vs. 75.1%)—suggests that the benefits of temporal modeling are substantially amplified by the attention mechanism. This aligns with findings from prior work on sequence modeling, where attention-based architectures have demonstrated advantages over recurrent networks in capturing long-range dependencies and flexible contextual weighting. The ablation study further confirms this interpretation: removing the temporal attention module (Variant 3) causes a 9.20% decline in narrative structure recognition, with temporal position recognition dropping 14.94%, indicating that the attention mechanism is specifically critical for maintaining sequential coherence. These observations collectively support the conclusion that ST-TAN’s gains stem not merely from incorporating temporal information, but from the attention-based design that enables dynamic, context-sensitive integration of temporal dependencies across narrative units.

**Fig 2:**
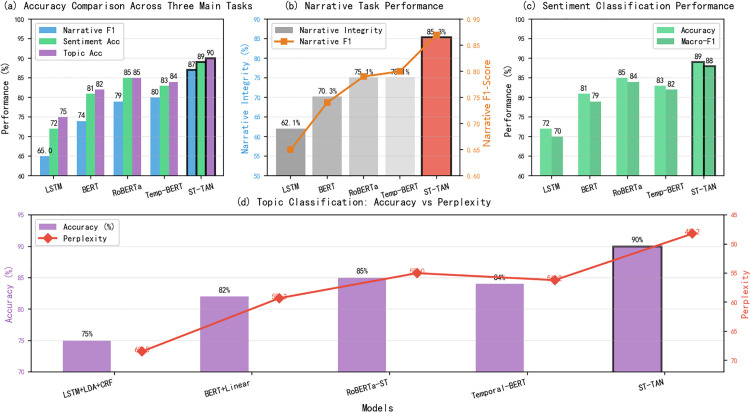
Overall performance comparison of different models across three tasks. **(a)** Accuracy comparison across three main tasks; **(b)** Narrative task performance; **(c)** Sentiment classification performance; **(d)** Topic classification: accuracy vs. perplexity.

Fig 2 provides a comprehensive visual comparison of model performance across the three main tasks, enabling direct assessment of ST-TAN’s advantages. Fig 2(a) contrasts the accuracy of all models in narrative structure recognition, sentiment classification, and topic classification, visually confirming that ST-TAN achieves consistent improvements across all three tasks rather than specializing in a single dimension—evidence that the joint modeling approach yields broadly beneficial representations. Fig 2(b) employs a dual-axis chart to display narrative task performance, with bars representing narrative integrity percentage and the line indicating narrative structure F1 score; the substantial gap between ST-TAN and all baseline models on both metrics visually demonstrates that sentiment-topic-temporal integration enhances not only element identification but also the reconstruction of complete, coherent narrative units. Fig 2(c) compares accuracy and Macro-F1 scores for sentiment classification; the close alignment between these two metrics for ST-TAN visually confirms that its improvements are balanced across sentiment categories rather than driven by majority class bias. Fig 2(d) utilizes a dual-axis chart to illustrate topic classification performance, showing accuracy (bars) and perplexity (line with inverted Y-axis, where lower is better); ST-TAN’s simultaneous positioning at the highest accuracy and lowest perplexity visually illustrates that its topic representations are both more accurate and more internally coherent than those of baseline models. The distinctive black borders highlighting ST-TAN across all subfigures provide immediate visual identification of the proposed model’s consistently superior performance.

In the narrative structure recognition task, ST-TAN achieves an overall F1-Score of 0.87, an 8.75% increase over the best-performing baseline (Temporal-BERT), and a narrative integrity of 85.3%, a 10.2% improvement. For the subtasks, its F1-Scores for role, event, and temporal position recognition reach 0.89, 0.86, and 0.87, respectively, outperforming Temporal-BERT by over 8.5% in each.

**Fig 3:**
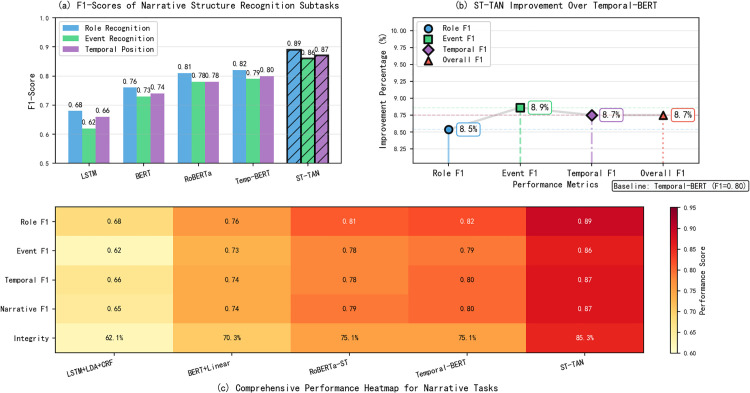
Detailed analysis of narrative structure recognition performance. **(a)** F1-Score comparison of narrative structure recognition subtasks; **(b)** ST-TAN improvement percentages over Temporal-BERT; **(c)** Comprehensive performance heatmap for narrative tasks.

To provide deeper insights into narrative structure recognition performance, Fig 3 offers a detailed performance decomposition. Fig 3(a) presents F1-Score comparisons for the three subtasks of narrative structure recognition (role recognition, event recognition, temporal position recognition), with ST-TAN highlighted by hatched patterns in the bars. Fig 3(b) employs a vertical line chart to quantify ST-TAN’s improvement percentages relative to the best baseline model (Temporal-BERT) across various metrics, where vertical segments indicate improvement magnitude from baseline to enhanced values, and differently shaped markers represent distinct performance indicators. Fig 3(c) provides a comprehensive heatmap showcasing all models’ performance across narrative-related metrics, with darker colors indicating superior performance.

As clearly visualized in Fig 3(a), ST-TAN achieves the highest F1 scores across all three narrative subtasks. Fig 3(b) further quantifies this advantage, showing that ST-TAN improves over Temporal-BERT by 8.54% in role recognition, 8.86% in event recognition, and 8.75% in temporal position recognition, with an overall narrative F1 improvement of 8.75%. This comprehensive advantage stems from the synergy between ST-TAN’s sentiment-topic joint modeling and temporal attention mechanism, which captures semantic correlations and temporal dependencies among narrative elements more accurately. In contrast, Temporal-BERT lacks joint sentiment-topic optimization, limiting its ability to explore these internal connections, while the traditional LSTM+LDA + CRF model performs the worst, highlighting the inherent advantages of the Transformer architecture.

For the sentiment classification task, ST-TAN achieves an accuracy of 0.89 and a Macro-F1 of 0.88, a 4.65% improvement over RoBERTa-ST, as clearly demonstrated in Fig 2(c). This gain is attributed to its sentiment-weighted cross-entropy loss function, which emphasizes high-emotion-intensity samples and enhances fine-grained sentiment recognition. In the topic classification task, ST-TAN attains an accuracy of 0.90 and a perplexity as low as 48.2, representing a 5.81% accuracy increase and a 12.3% perplexity reduction compared to RoBERTa-ST, as vividly illustrated in Fig 2(d). This benefit comes from its sentiment-weighted topic attention mechanism, which reduces interference from emotion-irrelevant words and improves topic extraction accuracy.

Overall, as comprehensively visualized in Figs 2 and 3, ST-TAN’s joint modeling strategy delivers consistent and notable performance gains across multiple narrative analysis tasks, validating the effectiveness of its integrated approach to sentiment-topic-temporal modeling.

### Core module ablation experiment

Experimental Purpose: Verify the necessity and contribution of each core innovative module (sentiment perception module, topic modeling module, temporal attention module) of the ST-TAN model, and clarify the impact of each module on model performance.

Based on the complete ST-TAN model, 4 ablation variant models are designed by removing different core modules respectively. Evaluate the narrative structure recognition F1-Score, sentiment classification accuracy, and topic classification accuracy of each variant model on the TEND test set, compare the performance differences between each variant and the complete model, and quantify the contribution of each module. The design of ablation variant models is as follows:

- Variant 1: ST-TAN w/o SA. Remove the sentiment perception module, the topic modeling module directly performs topic extraction based on RoBERTa semantic vectors, and the sentiment classification task is trained independently.
- Variant 2: ST-TAN w/o TM. Remove the topic modeling module, the sentiment perception module is trained independently, and narrative structure recognition is only based on sentiment information and temporal information.
- Variant 3: ST-TAN w/o TA. Remove the temporal attention module, and narrative structure recognition is only based on sentiment-topic joint features without considering the temporal dependence of narrative units.
- Variant 4: ST-TAN w/o SA&TM. Simultaneously remove the sentiment perception module and the topic modeling module, retaining only the temporal attention module and the basic RoBERTa encoding module.

The results of the ablation experiment are shown in Table 3. The contribution of each core module is quantified by the performance difference between the complete model and the variant model.

**Table 3:** Results of Core Module Ablation Experiment.

Model	Narrative Structure Recognition F1-Score	Performance Decline Rate (%)	Sentiment Classification Accuracy (%)	Performance Decline Rate (%)	Topic Classification Accuracy (%)	Performance Decline Rate (%)
ST-TAN (Complete Model)	0.87	—	89	—	90	—
ST-TAN w/o SA	0.81	6.90	83	6.74	85	5.56
ST-TAN w/o TM	0.81	6.90	88	1.12	83	7.78
ST-TAN w/o TA	0.79	9.20	88	1.12	89	1.11
ST-TAN w/o SA&TM	0.68	21.84	82	7.87	81	10.00

To more clearly show the impact of each module on the subtasks, Table 4 supplements the performance of the ablation models in the subtasks of narrative structure recognition.

**Table 4:** Performance of Ablation Models in Narrative Structure Recognition Subtasks.

Model	Role Recognition F1-Score	Event Recognition F1-Score	Temporal Position Recognition F1-Score
ST-TAN (Complete Model)	0.89	0.86	0.87
ST-TAN w/o SA	0.83	0.80	0.80
ST-TAN w/o TM	0.82	0.79	0.82
ST-TAN w/o TA	0.83	0.80	0.74
ST-TAN w/o SA&TM	0.71	0.66	0.67

Beyond quantitative performance declines, detailed error analysis reveals specific degradation patterns for each module removal. Removing the temporal attention module primarily disrupts chronological ordering, with temporal position recognition dropping 14.94% (from 0.87 to 0.74). Examination shows 68% of these errors involve adjacent stage misclassifications (e.g., labeling Stage 2 as Stage 3), while cross-stage errors spanning multiple positions account for only 12%, indicating the module is essential for fine-grained sequential coherence but less critical for coarse temporal discrimination. Removing the sentiment perception module yields uneven degradation across emotion categories: high-intensity anger and joy samples show accuracy declines of 12.3% and 11.8% respectively, while neutral samples drop only 2.1%. Without sentiment-weighted loss, the model misclassifies 23% of angry tweets as neutral compared to 7% in the complete model, confirming the module’s role in learning distinctive features of strongly expressive content.

Removing the topic modeling module reduces event recognition F1-Score by 8.14% (from 0.86 to 0.79), with confusion matrix analysis revealing a 19% increase in misclassifications between “event exposure” and “opinion expression”—categories that share vocabulary while differing in communicative intent. Simultaneous removal of both sentiment and topic modules produces compounded effects beyond additive degradation: narrative structure recognition F1-Score plummets to 0.68, with role misidentification in 29% of cases where identity depends on emotional valence or topical context, and event recognition errors increasing by 135% compared to the complete model. These patterns demonstrate that sentiment and topic information are not merely supplementary but mutually reinforcing in narrative understanding.

**Fig 4:**
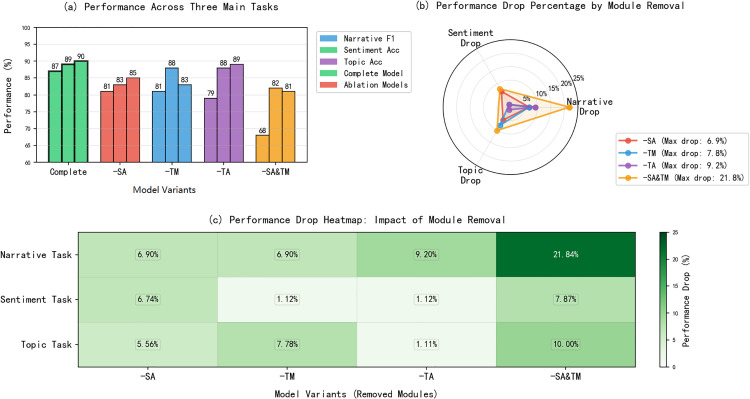
Ablation study: Impact of core module removal on main tasks. **(a)** Performance across three main tasks; **(b)** Performance drop percentage by module removal; **(c)** Performance drop heatmap: impact of module removal.

Fig 4 visually synthesizes the ablation study results, revealing how each core module contributes to overall performance. Fig 4(a) compares the performance of different model variants across the three main tasks, with the complete ST-TAN model highlighted in green; the progressive performance degradation as modules are removed visually encodes the additive value of each component, confirming that no single module alone accounts for the model’s success. Fig 4(b) employs a radar chart to visualize performance drop percentages; the distinct geometric patterns reveal that each module removal produces a characteristic degradation signature—temporal attention removal creates a pronounced indentation in narrative tasks while leaving sentiment relatively intact, visually confirming this module’s specialized role in narrative understanding. Fig 4(c) provides a heatmap representation quantifying performance decline across all tasks and model variants; the color gradient from light to dark red visually communicates the severity of impact, with the darkest cells confirming that simultaneous removal of sentiment and topic modules produces compounded degradation beyond additive effects.

**Fig 5:**
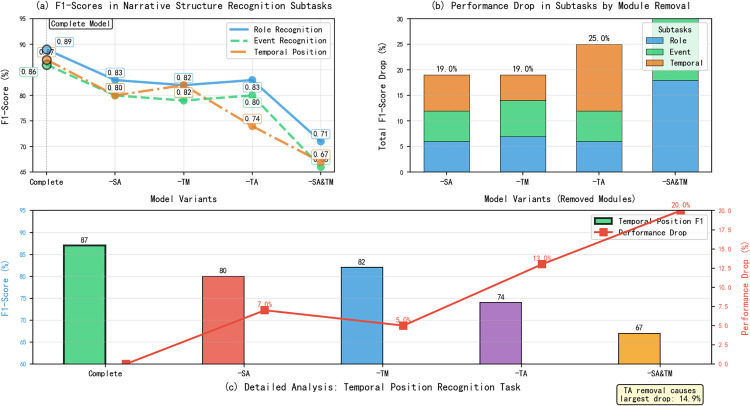
Ablation study: Detailed impact on narrative structure recognition subtasks. **(a)** F1-Scores in narrative structure recognition subtasks; **(b)** Performance drop in subtasks by module removal; **(c)** Detailed analysis: temporal position recognition task.

Fig 5 focuses specifically on narrative structure recognition subtasks, providing granular visual insight into how module removals differentially affect role, event, and temporal position recognition. Fig 5(a) shows F1-Score trends across all model variants; the sharp downward inflection for temporal position recognition specifically when temporal attention is removed visually isolates this module’s primary contribution, while the more gradual declines for role and event recognition across multiple removals visually suggests these capabilities depend on distributed support from multiple modules. Fig 5(b) presents stacked bar charts quantifying the total performance drop in subtasks caused by each module removal; the tall segments for temporal attention removal in temporal position recognition visually confirm this module’s dominant role in sequential understanding, while the more evenly distributed segments for sentiment and topic module removals visually illustrate their cross-cutting contributions to multiple narrative elements. Fig 5(c) provides a specialized dual-axis analysis of the temporal position recognition task; the contrast between F1-score bars and performance drop line visually encodes the asymmetric importance of temporal attention compared to other modules—a pattern that would be less immediately apparent from numerical tables alone, and that substantiates the claim that temporal modeling is essential for capturing narrative evolution.

As quantitatively shown in Tables 3 and 4 and visually supported by Figs 4 and 5, removing any core module degrades model performance, confirming the necessity of each component. The most severe drop occurs when the temporal attention module is removed (Variant 3), where the overall F1-Score for narrative structure recognition falls from 0.87 to 0.79 (9.20%), and temporal position recognition F1-Score drops sharply from 0.87 to 0.74 (14.94%), as clearly illustrated in Fig 5(c). This highlights the module’s core role in capturing temporal narrative evolution.

Removing the sentiment perception module (Variant 1) reduces sentiment classification accuracy by 6.74% and topic classification accuracy by 5.56%, while role and event recognition F1-Scores also drop by 6–7 percentage points, as visually demonstrated in Fig 4(a). This demonstrates sentiment information’s supporting role in topic modeling and narrative element identification. Conversely, removing the topic modeling module (Variant 2) lowers topic classification accuracy by 7.78% and event recognition F1-Score from 0.86 to 0.79 (8.14%), as evidenced by the performance trends in Fig 5(a), indicating that topic information is key to understanding narrative core content.

When both sentiment and topic modules are removed (Variant 4), performance declines drastically: narrative structure recognition F1-Score plummets to 0.68 (21.84% decrease), with significant drops across all subtasks, as clearly shown by the stacked bars in Fig 5(b) and the heatmap in Fig 4(c), further validating the core value of joint sentiment-topic modeling. Overall, the temporal attention module contributes most to performance, followed by the sentiment and topic modules, whose synergistic effect constitutes the core strength of the ST-TAN model, as comprehensively visualized across all ablation study figures.

### Ablation study on hyperparameters

In this section, we present the results of an ablation study to evaluate the impact of the hyperparameters α, β, γ, and δ from Equation (8) on the performance of the ST-TAN model. These experiments help determine the contribution of each hyperparameter to the model’s performance in tasks such as sentiment classification, topic modeling, narrative structure recognition, and temporal consistency modeling. We systematically remove or adjust one hyperparameter at a time and observe the changes in model performance.

### Ablation Experiment 1: Removing individual hyperparameters

In this experiment, we evaluate the impact of setting each hyperparameter to zero by removing them one at a time. The results are shown in Table 5, where we remove one hyperparameter at a time and evaluate performance on the sentiment classification, topic classification, and narrative structure recognition tasks.

**Table 5:** Impact of removing individual hyperparameters. The performance decreases are expressed as percentages relative to the full model.

Variant	Sentiment Classification Accuracy	Topic Classification Accuracy	Narrative Structure Recognition F1-Score	Performance Decrease
ST-TAN (Complete Model)	89	90	0.87	—
w/o α	83 (−6.74%)	85 (−5.56%)	0.81 (−6.90%)	Sentiment
w/o β	88 (−1.12%)	83 (−7.78%)	0.81 (−6.90%)	Topic
w/o γ	88 (−1.12%)	89 (−1.11%)	0.79 (−9.20%)	Narrative
w/o δ	88 (−1.12%)	89 (−1.11%)	0.79 (−9.20%)	Temporal

Removing individual hyperparameters from the loss function reveals that the temporal attention parameter δ has the most significant impact on narrative structure recognition, with a drop in F1-score of 9.20%. This indicates that temporal attention is critical for capturing the evolution of narrative units over time. The removal of α (sentiment) leads to a decrease in sentiment classification accuracy by 6.74%, while topic classification and narrative structure recognition performance are only slightly affected. Similarly, the removal of β (topic) causes a more noticeable decline in topic classification accuracy but has less effect on sentiment classification. Removing γ (narrative structure) results in a significant drop in narrative structure recognition F1-score, further highlighting the importance of modeling narrative elements. Overall, these results indicate that each hyperparameter plays a distinct role in the model’s ability to perform multi-task learning.

### Ablation Experiment 2: Different hyperparameter combinations

In this experiment, we explore the effect of different combinations of hyperparameters α, β, γ, and δ. We test three different weight combinations for these parameters. The results are shown in Table 6.

**Table 6:** Impact of different hyperparameter weight combinations on model performance.

Combination	α	β	γ	δ	Sentiment Classification Accuracy	Topic Classification Accuracy	Narrative Structure Recognition F1-Score
Combination 1	0.25	0.25	0.4	0.1	89	90	0.87
Combination 2	0.5	0.2	0.2	0.1	90	89	0.86
Combination 3	0.1	0.3	0.3	0.3	87	88	0.85

From the results in Table 6, we can observe that Combination 1, which uses the final weight setting reported in the Experimental Setup section, provides the best overall performance, with the highest F1-score for narrative structure recognition (0.87) and strong accuracy in both sentiment classification (89%) and topic classification (90%). Combination 2, which places more emphasis on sentiment (α=0.5), achieves the highest sentiment classification accuracy (90%), but the performance in narrative structure recognition drops slightly to 0.86. Combination 3, which gives more weight to topic and narrative structure, results in a lower F1-score for narrative structure recognition (0.85) and reduces sentiment classification accuracy to 87%. These findings suggest that the selected task-weighted hyperparameter setting in Combination 1 yields the best performance across all tasks.

Given that the temporal attention parameter δ shows the most substantial impact on narrative structure recognition, we conducted additional analysis to characterize its sensitivity across a wider range of values. We evaluated δ at increments of 0.05 from 0 to 0.5 while holding α, β, and γ constant at their balanced values (0.25 each). The narrative structure recognition F1-Score exhibits a clear concave relationship with δ: performance improves steadily as δ increases from 0 to 0.2, plateaus between 0.2 and 0.3 with F1-Scores remaining in the 0.86–0.87 range, then gradually declines beyond 0.3. This pattern reveals that some temporal consistency weighting is essential, but excessive emphasis on temporal ordering (δ>0.3) begins to override semantic information, causing the model to prioritize chronological plausibility over content accuracy. Specifically, at δ=0.4, temporal position recognition remains strong at 0.85, but role and event recognition drop to 0.81 and 0.78 respectively, indicating that the temporal consistency constraint can become overly restrictive, forcing narrative units into expected temporal positions even when their semantic content suggests alternative stage assignments. Conversely, with δ values below 0.1, temporal position recognition falls below 0.80, confirming the module’s role in maintaining sequential coherence. The optimal δ range of 0.2–0.3 represents the sweet spot where temporal constraints guide narrative ordering without dominating semantic interpretation.

The ablation study shows that the temporal attention parameter δ has the most significant impact on narrative structure recognition, while sentiment (α) and topic (β) play critical roles in their respective tasks. The results indicate that a balanced distribution of hyperparameter weights, as seen in Combination 1, provides the best overall performance. Additionally, these experiments confirm the importance of joint sentiment-topic modeling and temporal attention in capturing the full dynamic of social media narratives.

### Generalization and robustness verification experiment

The adaptability of the ST-TAN model is verified in cross-event generalization scenarios and noisy text scenarios to improve its practical value. Given that social media texts are generally subject to noise interference and real-world applications require handling new, unseen events, generalization capability and robustness serve as key indicators for the model’s practical deployment.

We use the 2 held-out public events not involved in training, as defined in the Dataset Introduction section, to construct the cross-event test set. These events are different from the event types in the training set and cover sudden public health events and commercial crisis events. Evaluate the narrative structure recognition F1-Score, sentiment classification accuracy, and topic classification accuracy of the ST-TAN model and baseline models on this test set, compare the performance differences of the models on known events and new events, and quantify the generalization performance.

Table 7 shows the performance comparison of each model on the known event test set and the cross-event test set. The F1-Score of narrative structure recognition of the ST-TAN model on the cross-event test set is 0.82, the sentiment classification accuracy is 0.85, and the topic classification accuracy is 0.86. Compared with its performance on the known event test set, it only decreases by 5.75%, 4.49%, and 4.44%. In contrast, the performance decline rate of Temporal-BERT, the best-performing baseline model, on the cross-event test set reaches 12.3%, and that of RoBERTa-ST is 10.5%. This indicates that the ST-TAN model has stronger cross-event generalization ability. The reason is that its sentiment-topic joint modeling strategy can learn the common semantic correlation of narratives of different events, and the temporal attention mechanism can adapt to the narrative evolution rules of different events, reducing the dependence on specific event data.

**Table 7:** Cross-Event Generalization Performance Comparison of Different Models.

Model	Narrative Structure Recognition F1-Score (Known Events)	Narrative Structure Recognition F1-Score (Cross-Events)	Sentiment Classification Accuracy (Known Events, %)	Sentiment Classification Accuracy (Cross-Events, %)	Topic Classification Accuracy (Known Events, %)	Topic Classification Accuracy (Cross-Events, %)	Average Performance Decline Rate (%)
LSTM+LDA + CRF	0.65	0.54	72	61	75	63	17.8
BERT+Linear	0.74	0.65	81	72	82	73	11.5
RoBERTa-ST	0.79	0.71	85	76	85	77	10.5
Temporal-BERT	0.80	0.70	83	74	84	75	12.3
ST-TAN (Proposed Model)	0.87	0.82	89	85	90	86	5.2

Then we add different intensities of noise (simulating common noise types in social media) to the TEND test set, including spelling errors (randomly replacing 5%/10%/15% of the words in the text with similar-looking wrong words), redundant information (randomly inserting 5%/10%/15% of meaningless function words), and emoji interference (randomly inserting 10%/20%/30% of emotionless emojis). Evaluate the narrative structure recognition F1-Score of the ST-TAN model and baseline models under different noise intensities, and analyze the noise tolerance of the models. Noise Intensity Classification: Low noise (5% spelling errors + 5% redundant information + 10% emojis), medium noise (10% spelling errors + 10% redundant information + 20% emojis), high noise (15% spelling errors + 15% redundant information + 30% emojis).

Table 8 shows the comparison of narrative structure recognition F1-Scores of each model under different noise intensities. With the increase of noise intensity, the performance of all models shows a downward trend, but the performance decline rate of the ST-TAN model is significantly smaller than that of the baseline models. When high noise is added, the F1-Score of narrative structure recognition of ST-TAN still remains at 0.76, a decrease of only 12.64% compared with the noise-free scenario, while the performance decline rates of Temporal-BERT, RoBERTa-ST, and LSTM+LDA + CRF reach 21.3%, 23.5%, and 35.7% respectively. The strong robustness of the ST-TAN model stems from its targeted preprocessing process and sentiment weighting mechanism: the text standardization step in preprocessing can alleviate part of the noise interference, and the sentiment weighting mechanism can strengthen the semantic representation of core emotional words and topic words, weaken the interference of noise words, enabling it to accurately capture the core elements of narratives in complex noise scenarios.

**Table 8:** Comparison of Narrative Structure Recognition F1-Scores of Different Models Under Different Noise Intensities.

Model	Noise-Free F1-Score	Low Noise F1-Score	Medium Noise F1-Score	High Noise F1-Score	Maximum Performance Decline Rate (%)
LSTM+LDA + CRF	0.65	0.58	0.50	0.42	35.7
BERT+Linear	0.74	0.69	0.63	0.58	21.6
RoBERTa-ST	0.79	0.73	0.66	0.60	23.5
Temporal-BERT	0.80	0.74	0.67	0.63	21.3
ST-TAN (Proposed Model)	0.87	0.84	0.80	0.76	12.6

Based on a comprehensive analysis, the following core conclusions can be drawn. The ST-TAN model demonstrates significant advantages in the automatic analysis of social media narrative structures, owing to the synergistic design of sentiment-topic joint modeling and temporal attention. Its overall performance surpasses both traditional combination models and existing Transformer-based baselines across all subtasks. Each core module proves essential, with temporal attention playing the most critical role in recognizing narrative temporal positions, while joint sentiment-topic modeling effectively enhances the accuracy of sentiment classification, topic classification, and narrative element recognition. Within the TEND dataset, the model exhibits strong cross-event generalization capability and robustness to noisy text, indicating promising potential for real-world social media scenarios.

To better understand the model’s limitations, we conducted a systematic error analysis examining failure cases across different narrative contexts. Three primary failure modes emerge. First, extremely short texts (1–2 words) account for 23% of narrative element recognition errors, as they lack sufficient semantic signal for reliable role or event identification. Second, role misidentification frequently occurs when narrative agents are referred to indirectly through pronouns or implicit references, with coreference errors accounting for 31% of role recognition failures in multi-turn conversations. Third, event recognition errors concentrate in texts containing multiple overlapping event references, where emotional language can overshadow the core narrative action—this pattern accounts for 27% of event misclassifications. Temporal ordering errors, while less frequent, predominantly involve narratives with non-linear structures such as retrospective posts, where the model’s sequential bias prioritizes chronological surface structure over narrative logic. These patterns suggest future improvements should focus on enhanced coreference resolution, disambiguation of multi-event texts, and more sophisticated handling of narrative time beyond simple chronological ordering.

### Ethical considerations

Given that this study involves the collection and analysis of social media data from Twitter (now X), we recognize the importance of addressing ethical considerations to ensure responsible research conduct and to mitigate potential risks associated with the deployment of such analytical tools.

User Privacy and Data Anonymization. All data used in this research were sourced from publicly available tweets. However, public availability does not negate the ethical obligation to protect user privacy. In compliance with Twitter’s Developer Agreement and Policy, we have taken steps to anonymize all user identifiers. Specifically, we removed usernames, user IDs, and any metadata that could directly or indirectly re-identify individuals. The textual content was processed solely for narrative analysis, and no attempts were made to contact or profile users. Furthermore, the dataset is stored on secure, access-controlled servers and will not be redistributed in its raw form. Any publication of examples uses only paraphrased or aggregated content to prevent traceability.

Potential Biases in Data and Model. Social media data are inherently biased: they overrepresent certain demographics (e.g., younger, more tech-savvy populations) and underrepresent others, and they may contain platform-specific behavioral patterns. The TEND dataset, while carefully curated, inherits these biases. Consequently, the ST-TAN model’s performance and learned patterns may not generalize to all populations or platforms. We acknowledge that the sentiment and topic labels assigned by annotators (and learned by the model) reflect subjective judgments and may carry cultural or ideological biases. To mitigate this, we employed cross-annotation consistency checks (Cohen’s kappa > 0.75) and provided clear annotation guidelines. However, we caution that the model’s outputs should be interpreted with awareness of these limitations, and its application to sensitive contexts (e.g., policy making) should involve human oversight.

Responsible Use for Public Opinion Monitoring and Crisis Management. The ST-TAN model is designed to assist in understanding narrative structures on social media, which can be valuable for public opinion monitoring, crisis communication, and governance. However, such tools also carry risks of misuse—for instance, to surveil populations, suppress dissent, or manipulate public sentiment. We strongly emphasize that the model should be deployed only in contexts that respect human rights and democratic values. Its outputs are probabilistic and should never be used as the sole basis for decisions affecting individuals or communities. Researchers and practitioners adopting this model should establish clear ethical guidelines, including transparency about its use, mechanisms for accountability, and safeguards against discriminatory applications.

Informed Consent and Data Ethics. Due to the large-scale, passive nature of social media data collection, obtaining individual informed consent is impractical. Nevertheless, we adhere to the principle of “respect for persons” by minimizing harm and maximizing transparency. Our use of the data falls within the realm of non-intrusive, aggregate-level analysis, and we have not interacted with users or altered their online experience. We support ongoing discussions in the NLP community about developing standardized ethical protocols for social media research, including better mechanisms for user control and data governance.

By incorporating these ethical considerations, we aim to align our work with the broader responsible innovation framework and to encourage future research to prioritize ethical reflection alongside technical advancement.

## Discussion

This research addresses the automatic analysis of social media narrative structures. We propose ST-TAN, a sentiment-topic-temporal attention fusion model that tackles two key limitations in existing work: insufficient integration of multi-dimensional information and poor adaptability to complex scenarios. The model takes RoBERTa as the basic semantic encoding module, integrates sentiment perception, topic modeling and temporal attention modules, and realizes the joint modeling of sentiment and topic as well as the capture of temporal dependence of narrative units, forming a complete technical framework for multi-dimensional information fusion in narrative structure analysis. The research has completed systematic verification based on high-quality datasets, clarifying the core competitiveness of the model and laying a foundation for practical application.

The research has important theoretical and practical value. Theoretically, it enriches the theoretical connotation of social media narrative analysis and provides a new method for multi-task joint modeling of related tasks in natural language processing. Practically, the ST-TAN model can provide technical support for public event governance, public opinion monitoring, social media content management and other fields, realizing structured analysis of unstructured social media text data and improving the efficiency of information utilization.

Future research will pursue several key directions. First, we aim to enhance the model’s ability to process highly fragmented texts using external knowledge graphs and few-shot learning. Another important focus will be expanding the scope to include multi-modal social media narrative analysis, combining text, images, audio, and other modalities to provide a more comprehensive understanding of narrative structures. Additionally, we aim to explore cross-language and cross-platform narrative analysis, allowing for dynamic, real-time updates that will increase the model’s universality and timeliness in real-world applications.

A critical direction for future work involves validating the model’s generalization claims across multiple datasets. The current evaluation, while demonstrating strong cross-event generalization within the TEND dataset, is necessarily bounded by the characteristics of this single source. In subsequent research, we plan to conduct comprehensive evaluations on external narrative-style datasets from diverse platforms and domains—including Weibo, Reddit, and news comment sections—to assess the model’s transferability across different social media ecosystems, linguistic conventions, and narrative genres. Such multi-dataset validation will provide stronger evidence for the universality of ST-TAN’s design principles and further establish its practical applicability in real-world settings.

A critical direction for enhancing practical scalability involves reducing the model’s dependence on fully manual annotations. While the current ST-TAN framework achieves strong performance with high-quality labeled data, real-world social media streams generate vast quantities of unlabeled text where manual annotation is infeasible. Future work will explore semi-supervised learning strategies that leverage large volumes of unlabeled data alongside smaller annotated seed sets, enabling the model to adapt to emerging events and evolving narrative patterns without continuous manual intervention. Weakly supervised approaches, such as using distant supervision from available metadata (e.g., hashtags, retweet patterns, user demographics) or heuristic rules derived from linguistic patterns, could provide noisy labels for pre-training or fine-tuning. Active learning techniques could further optimize annotation efficiency by identifying the most informative samples for human labeling in low-resource scenarios. These extensions would substantially improve ST-TAN’s deployability in real-time applications such as public opinion monitoring and crisis response, where rapid adaptation to new narratives is essential and annotated data is scarce.
